# Current understanding of tyrosine kinase BMX in inflammation and its inhibitors

**DOI:** 10.4103/2321-3868.135483

**Published:** 2014-07-28

**Authors:** Le Qiu, Fei Wang, Sheng Liu, Xu-Lin Chen

**Affiliations:** Department of Burns, First Affiliated Hospital of Anhui Medical University, 218 Jixi Road, Hefei, Anhui, 230022 China

**Keywords:** BMX, inflammation, tyrosine kinase

## Abstract

Tec family kinases, which include tyrosine kinase expressed in hepatocellular carcinoma (TEC), Bruton’s tyrosine kinase (BTK), interleukin (IL)-2-inducible T-cell kinase (ITK), tyrosine-protein kinase (TXK), and bone marrow tyrosine kinase on chromosome X (BMX), are the second largest group of non-receptor tyrosine kinases and have a highly conserved carboxyl-terminal kinase domain. BMX was identified in human bone marrow cells, and was demonstrated to have been expressed in myeloid hematopoietic lineages cells, endothelial cells, and several types of cancers. Significant progress in this area during the last decade revealed an important role for BMX in inflammation and oncologic disorders. This review focuses on BMX biology, its role in inflammation and possible signaling pathways, and the potential of selective BMX inhibitors.

## Introduction

The response of cells to extracellular stimuli is in part mediated by a number of intracellular kinases. Tec family kinases, as the second largest group of non-receptor tyrosine kinases, are of critical importance to the biology of lymphocytes and other cell lineages derived from the bone marrow.[1] Five distinct subgroups within the Tec kinase family have been described. These include (1) tyrosine kinase expressed in hepatocellular carcinoma (TEC), (2) Bruton’s tyrosine kinase (BTK), (3) interleukin (IL)-2-inducible T-cell kinase (ITK), (4) tyrosine-protein kinase (TXK), and (5) bone marrow tyrosine kinase on chromosome X (BMX).[2] Tec family kinases have been the focus of immunological interest ever since their discovery.

**Table 1:** 

Access this article online	
**Quick Response Code**:	**Website**: www.burnstrauma.com
	**DOI**: 10.4103/2321-3868.135483

While BTK, ITK and TXK show selective expression in cells of bone marrow origin,[3] the expression patterns of BMX and TEC are broader and extends to certain normal somatic cells — such as the cardiac endothelium as a response to ischemia and pressure overload.[4] Specifically, BMX is expressed in hematopoietic cells of the myeloid lineage like granulocytes and monocytes.[5,6] Besides, BMX expression has also been demonstrated in glioblastoma cancer stem cells and several solid tumors, such as prostate and breast cancer. BMX has been suggested to have a role in differentiation, motility and cell survival.[7] The endothelial cells, granulocytes and monocytes play critical roles in the inflammation. This review will examine BMX biology, its role in inflammation and possible signaling pathway, and the potential of selective BMX inhibitors.

## Structure and function of BMX

**Figure 1:**
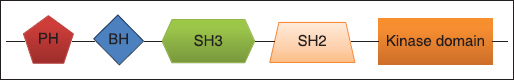
Schematic representation of bone marrow tyrosine kinase on chromosome X (BMX) structural domains. PH = pleckstrin homology, BH = BTK homology, SH = Src homology.

Like many other kinase families, members of the Tec kinase have a typical array of regulatory domains and a highly conserved carboxyl-terminal kinase domain [Figure 1].[8] As such, BMX has Src homology (SH)3 and SH2 domains and a carboxyl-terminal kinase domain.[9] The aminoterminus contains a membrane localization module that is a characteristic feature of the TEC kinases and sets them apart from other non-receptor tyrosine kinases.[10] An amino-terminal pleckstrin homology (PH) domain, which binds to phosphatidylinositols during the process of membrane localization,[11] is followed by a zinc-binding BTK homology (BH) motif and a proline-rich region, which shows a low degree of conservation to the other family members.[7] The SH2, SH3 and BH domains all mediate inter- and intramolecular protein interactions that are likely to regulate kinase activity and substrate access.[7]

BMX was the latest identified one among the 5 human TEC kinases. In 1994, the human BMX gene was first identified and cloned in bone marrow cells by Tamagnone *et al.*[12] The BMX gene is located in chromosomal band X p22.2 between the DXS197 and DXS207 loci.[12] The BTK gene, the closest relative of BMX, is also located in chromosome X. The BMX gene encodes a protein with 675 amino acids, of which 70% are identical with BTK.[12] Mutations in BTK gene are responsible for X-linked agammaglobulinemia (XLA) in humans or X-linked immunodeficiency (XID) in mice.[8] However, diseases-associated BMX gene mutations have not been described yet.

## BMX in inflammation

Inflammation is a necessary and rapid, yet coordinated response that is induced by microbial infection or tissue injury.[13] Triggers capable of inducing an inflammatory response include tissue damage and infection by pathogenic and nonpathogenic microbes.[13] Undue prolongation of inflammation can be very destructive or even initiate the systemic inflammatory response syndrome, multiple organ failure and death.[14] The inflammatory cytokines, which affect various and numerous physiologic activities, play a significant role in the pathogenesis of inflammation. In the previous studies, tumor necrosis factor (TNF)-α, IL-1β and IL-6 have been demonstrated to be the core of the cytokine-network and play a critical role in the inflammatory response.[15,16] During the immune response, the cytokine IL-8 functions as a potent neutrophil attractant and activator leads to the recruitment of neutrophils from blood, penetration of these cells through the vessel wall, and their directed migration to inflammatory sites and contributes to the advance of inflammation by releasing superoxide anion, matrix metalloproteinase, leukotriene B(4) and platelet-activating factor.[17–19]

There is emerging evidence that non-receptor tyrosine kinase BMX is involved in the pathogenesis of inflammatory disorders, such as rheumatoid arthritis (RA).[7,20,21] An siRNA against BMX-inhibited lipopolysaccharide (LPS)-induced IL-6 secretion in synovial fibroblasts.[22] In macrophages and synovial fibroblasts from RA patients, overexpression of BMX mediates an increase in LPS-induced stabilization of the IL-6 mRNA.[20,21] In the absence of LPS, overexpression of BMX failed to induce IL-6 mRNA expression. Further study revealed that transient depletion of BMX strongly reduced secretion of IL-8 in human fibroblasts stimulated by TNF-α and IL-1β.[7] In neuronal injury induced by H_2_O_2_ or ischemia, BMX is activated and suppressing BMX activity protects against neurodegeneration.[23] Altogether, an essential role for the tyrosine kinase BMX in cytokine signaling and inflammation has been established.

The stimulation of Toll-like receptor (TLR)4 by LPS induces the release of critical pro-inflammatory cytokines that result in systemic inflammation and sepsis.[16,24] TLR4 signaling has been divided into MyD88-dependent and MyD88-independent pathways. Signaling through the MyD88-dependent pathway leads to the activation of p38 mitogen-activated protein kinase (MAPK), c-Jun NH(2)-terminal kinase (JNK), and nuclear factor-κB (NF-κB).[25,26] Such signaling activation consequently lead to the release of pro-inflammatory cytokines including TNF-α and IL-1β.[27,28]

**Figure 2:**
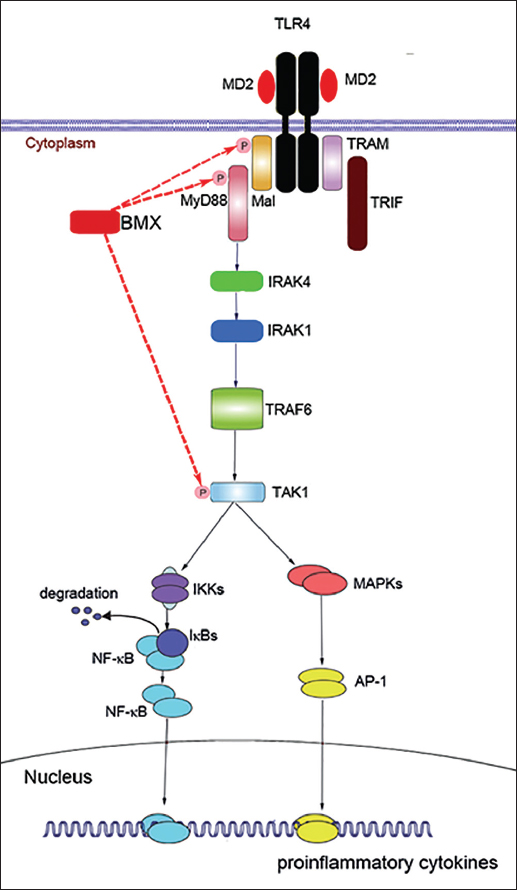
Role of bone marrow tyrosine kinase on chromosome X (BMX) in Toll-like receptor (TLR)4 signaling. BMX has been reported to interact with adaptor molecules (MyD88, or Mal) and transforming growth factor (TGF)-β activated kinase (TAK)1, leading to the activation of the NF-κB and MAPK-signaling pathways. This process results in inflammatory cytokine production. MD = myeloid differentiation protein, TRIF = Toll/interleukin-1 receptor domain-containing adaptor protein inducing interferon-β, TRAM = TRIF-related adaptor molecule, IRAK = interleukin-1 receptor-associated kinase, TRAF = TNF receptor-associated factor, MAPK = mitogen-activated protein kinases, IKK = inhibitor kappa B kinase, AP = activator protein.

An siRNA against BMX strongly reduces secretion of IL-8 in cell lines and HUVECs stimulated by TLR4 agonist, suggesting that BMX could play a role in inflammatory-signaling pathways.[7] Study performed on RA synovial fibroblasts demonstrated that BMX is activated by TLR agonists and that it functionally and physically interacts with the components of the MyD88-dependent TLR pathway MyD88 and Mal as shown by co-immunoprecipitation.[22] More specifically, BMX was required for phosphorylation of p38 MAPK and JNK, as well as activation of NF-κB.[7,11,29] An epistasis analysis indicated that BMX functionally regulates the complex transforming growth factor (TGF)-β activated kinase (TAK)1-binding protein complex.[7] The possible role of BMX in TLR4 signaling is shown in Figure 2. Studies performed on COS cells and HEK293 cells revealed that BMX induced the tyrosine phosphorylation and DNA binding activity of all the Stat factors tested, including STAT1, STAT3 and STAT5.[30] Further study demonstrated that BMX is a critical mediator of Src-induced cell transformation and STAT3 activation.[31,32] However, the upstream localization of BMX kinase relative to the adaptor molecules and the exact mechanism of BMX-dependent regulation of cytokine gene expressions warrants further studies.

## BMX kinase inhibitors

As shown in the previously mentioned data, BMX is an important molecule involved in the inflammatory processes. Modulating BMX activity might control the excessive inflammatory response. Prerequisite for the treatment of inflammation by modifying BMX activity is to use a specific, non-toxic inhibitor.[33]

**Figure 3:**
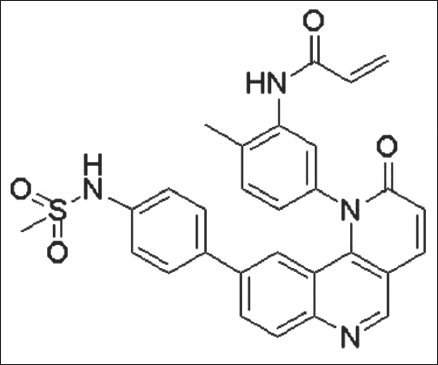
The chemical structure of BMX-IN-1, an irreversible BMX inhibitor. [Figure is from *Liu F, et al. ACS Chem Biol 2013;8(7):1423-8.*][33]

In order to develop irreversible BMX inhibitors, Liu and his colleagues developed compounds capable of targeting cysteine (Cys) 496. Kinome-wide sequence alignment reveals that all 5 TEC family kinases have the conserved cysteine. Dr. Liu introduced an electrophilic acrylamide moiety targeting Cys 496 and first successfully synthesized a selective BMX inhibitor, called BMX-IN-1.[34] The chemical structure is shown in Figure 3. In RV-1 cells, 1 µM of BMX-IN-1 is sufficient to inhibit BMX autophosphorylation.[34]

Ibrutinib (PCI-32765), a reported irreversible inhibitor of BTK, is also a potent inhibitor of BMX and other TEC family kinases.[35,36] Primary data showed that ibrutanib is beneficial in models of arthritis and patients with relapsed or refractory chronic lymphocytic leukemia.[36,37]

Whether these inhibitors could provide significant beneficial effects in inflammatory diseases remains to be established.
